# Monkeypox transmission following exposure in healthcare facilities in nonendemic settings: Low risk but limited literature

**DOI:** 10.1017/ice.2022.152

**Published:** 2022-07

**Authors:** Kimon C. Zachary, Erica S. Shenoy

**Affiliations:** 1 Regional Emerging Special Pathogens Treatment Center, Massachusetts General Hospital, Boston, Massachusetts; 2 Infection Control Unit, Massachusetts General Hospital, Boston, Massachusetts; 3 Division of Infectious Diseases, Department of Medicine, Massachusetts General Hospital, Boston, Massachusetts; 4 Harvard Medical School, Boston, Massachusetts

## Abstract

Transmission risk of monkeypox in healthcare settings outside endemic regions has not been well defined. A rapid review of the literature, including cases outside monkeypox-endemic regions from 2000 to 2022 identified a single reported case of transmission. Available literature is limited by nonstandardized exposure definitions and limited detail describing exposures.

The current monkeypox outbreak in multiple countries outside endemic regions has highlighted the limited knowledge of risk of transmission from infected patients in healthcare settings to others, including patients and healthcare personnel (HCP). Understanding the risk of transmission, and specifically the types of exposures in healthcare settings that may confer higher risk, is essential for infection prevention and control strategies, as well as to inform recommendations for postexposure monitoring and postexposure prophylaxis (PEP). Transmission to HCP in endemic settings is well described,^1,2^ but to date it appears to be rare in well-resourced settings. In this rapid literature review, we identified published studies of cases of monkeypox outside endemic regions where nosocomial exposure was described. We found a single documented transmission event; however, variable definition of exposure and limited specific details of the circumstances leading to exposure highlight the need for additional efforts to define and characterize exposures to monkeypox in healthcare settings.

## Methods

We performed searches of PubMed and Embase in May 2022, supplemented by a manual search by a medical librarian. No limit on language was imposed. The search combined the concepts of monkeypox, disease transmission, and humans. It excluded studies exclusively set in endemic regions, literature reviews, and studies published prior to the year 2000. The Embase search strategy is available upon request, and the PubMed search was conducted using the following strategy: ((monkeypox[title] OR “monkey pox”[title] OR “Monkeypox”[Mesh] OR “Monkeypox virus”[Mesh]) AND (“transmission”[Subheading] OR epidemiology[subheading] OR “Disease Transmission, Infectious”[Mesh] OR transmit*[tw] OR spread[tw] OR outbreak*[tw] OR cases[tw] OR case[tw] OR imported[tw]) AND (humans[mesh] OR “health personnel”[mesh] OR human[tw] OR humans[tw] OR person[tw] OR persons[tw] OR traveler[tw] OR travelers[tw] OR travelled[tw] OR traveled[tw] OR patient[tw] OR patients[tw] OR healthcare[tw] OR “health care”[tw]) NOT (“Africa”[Mesh] NOT (“Americas”[Mesh] OR “Asia”[Mesh] OR “Europe”[Mesh] OR “Oceania”[Mesh]))) AND 2000/01/01:2022/12/31[dp] NOT review[pt].

Our review was restricted to studies that identified nosocomial exposures and subsequent management. Studies that described monkeypox cases in ambulatory clinics, emergency departments, and inpatient settings that did not comment on HCP exposure were excluded. For each publication, we extracted the definition of exposures, when provided, the total number of HCP exposed, and assessment of monkeypox infection among those exposed (symptom monitoring or serological analyses).

## Results

**Fig. 1.**
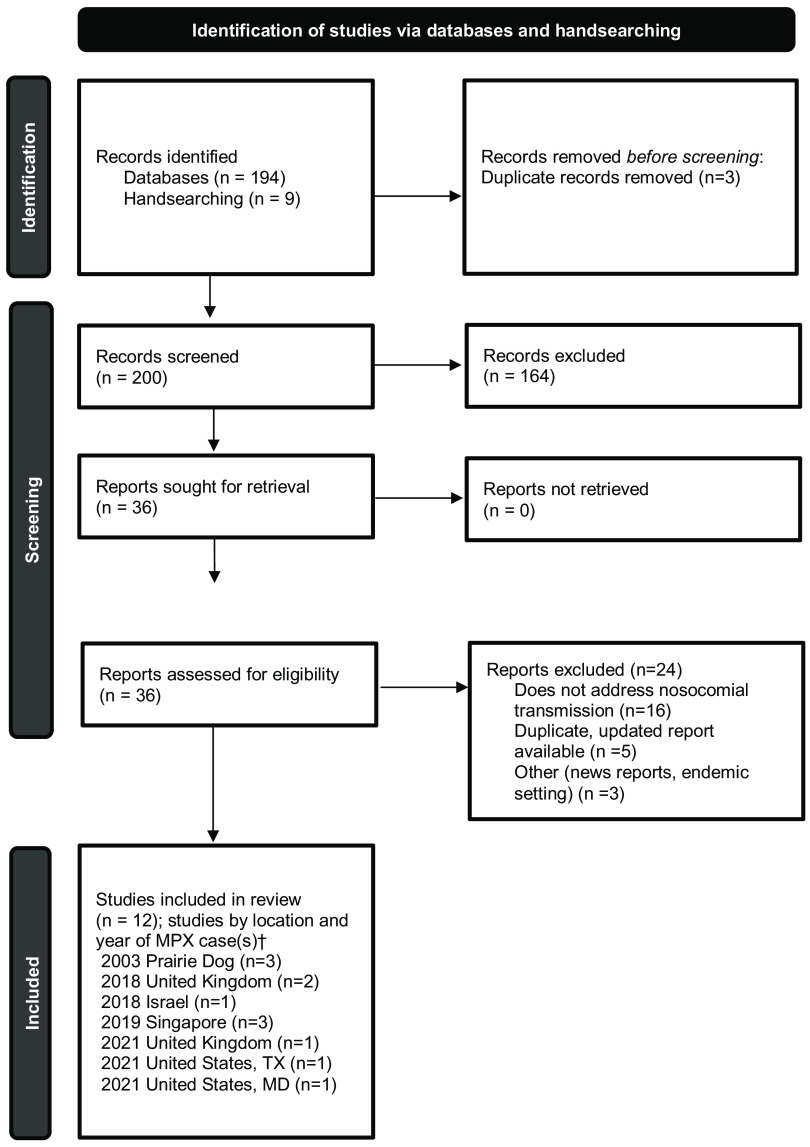
Preferred reporting items for systematic reviews and meta-analyses **(**PRISMA) flow chart. Identification of studies, screening, and inclusion criteria are provided. The numbers of studies are listed. ^†^Multiple studies may have been included describing the same case(s).

The search yielded 194 publications, and an additional 9 studies were manually selected, for a total of 203 studies for screening. After the removal of 3 duplicates, 200 studies were screened, of which 164 were excluded. Among 36 studies assessed for eligibility through full-text review, 24 were excluded, leaving a total of 12 studies for inclusion (Fig. 1).^3^ Of the 12 studies included, multiple studies described the same cases or outbreak and were combined in the analysis when details from >1 publication were within the scope of review.

Between 2000 and 2022, not including the current outbreak, we identified cases of monkeypox that were diagnosed outside endemic regions and were cared for in healthcare settings that reported exposure of HCP and subsequent evaluation. These cases were identified as part of the 2003 prairie dog–associated outbreak in the United States and, between 2018 and 2021, from outbreaks in the United Kingdom, Israel, Singapore, and the United States, in travelers returning from endemic areas (Table 1).

**Table 1.** Healthcare-Associated Monkeypox Exposures, Management, and Risk of Transmission in Nonendemic Countries, 2000–2022

Year	Country	Description	Definition of HCP Exposure	Methods to Assess for Monkeypox Infection Following Exposure	Outcomes, Including Risk Assessment, Nosocomial Transmission, and Administration of Postexposure Prophylaxis (PEP)	Reference
2003	United States, Illinois, Indiana, Kansas, Missouri, Ohio, and Wisconsin	Describes exposure investigation after 3 monkeypox patients identified as part of the 2003 prairie dog outbreak were admitted to hospital	HCP entered 2 m radius of the patient	Symptom monitoring; serology	81 HCP exposed; 57 (70%) participated; 40 of 57 (70%) had ≥ 1 unprotected exposure defined as not using gloves, gown, and either surgical mask or N95 respirator; no symptoms reported; 31 (54%) of 57 reported some history of prior smallpox vaccination; 1 HCP had antiorthopoxvirus IgM detected and had been vaccinated <6 mo prior; no transmissions reported.	Fleischauer et al^8^
		Summary of the 2003 prairie dog outbreak with a total of 71 cases (suspected and confirmed), including use of pre-exposure prophylaxis and PEP in HCP	Not provided	Not provided	Details of patient to HCP exposures not provided; among 30 individuals (HCP and non-HCP) in whom smallpox vaccine was administered, 2 HCP received vaccine as part of pre-exposure prophylaxis, and 10 HCP as PEP; no transmissions reported.	Gross^9^ *MMWR* update^10^
2018	United Kingdom	Two travel-related cases; case 1 initially presented to general practitioner and later admitted to hospital; case 2 presented to an ED and was admitted to hospital	Different criteria used for case 1 (southwest England) and case 2 (northwest England)	Symptom monitoring; HCP who developed symptoms were directed to phone their designated public health and to stop working until they were assessed by the imported fever service (IFS).	High risk: 5 (5 received PEP); intermediate risk: 125 (84 received PEP); low risk: 158 (0 received PEP); **1 HCP in high-risk category developed infection**; HCP described as having changed bedding without respiratory protection during period when monkeypox patient had active lesions prior to isolation; received PEP with attenuated nonreplicating vaccinia vaccine >4 d after exposure	Vaughan et al^4^ Vaughan et al^11^
2018	Israel	Travel-related case; patient presented to an ED and admitted to hospital	Not provided	No details provided; only that all contacts were followed up for 21 d; no transmission was detected	11 HCP identified as exposed without details; all offered PEP with one HCP vaccinated; no transmissions reported.	Erez et al^12^
2019	Singapore	Travel-related case; patient presented to an ED and admitted to hospital	Due to up front suspicion for monkeypox, all HCP were wearing PPE; Ambulance HCP: N95 respirator, gown, gloves, no eye protection; HCP at hospital: N95 respirator, gown, gloves, eye protection); patient placed in AIIR	Symptom monitoring	No HCP were exposed; all asymptomatic; no transmissions; 27 HCP identified, but all with appropriate PPE.^13^ Contact tracing of community exposures including 23 “close contacts” within 2 m of the patient for >30 min or had physical contact with patient or surfaces or materials contaminated by secretions (19 individuals who attended the same conference and 4 hotel staff) and 8 lower risk without definition of lower risk; 14 of 22 close contacts (1 of 23 had left the country) received PEP with with live, attenuated vaccinia virus (2 had contraindications and 6 declined)	Kyaw et al^13^ Ng et al^14^ Yong et al^15^
2021	United States, Texas	Travel-related case; patient presented to the ED and admitted to hospital	High, intermediate, low/uncertain, no risk; reports on mostly nonhealthcare exposures; based on CDC published exposure guidelines (since updated)	Symptom monitoring	High: 0; intermediate: 31 non HCF; 3 lab; low/uncertain: 146 non HCF; 43 HCP (care with gown, gloves, eye protection, N95 respirator or equivalent); no transmissions reported.	Rao et al^16^
2021	United States, Maryland	Travel-related case; patient presented to the ED and admitted to hospital	High, intermediate, low/uncertain, no risk; reports on mostly nonhealthcare exposures; based on CDC published exposure guidelines (since updated)	Symptom monitoring	40 HCP identified as contacts; none in high-risk group according to contemporary CDC guidelines; no PEP administered; no transmissions reported.	Costello et al^17^
2021	United Kingdom	Travel-related case resulting secondary transmission to 2 family members; case 1 presented to an ED and was initially discharged but then admitted to hospital the next day; entire household eventually admitted for observation after case 2 (child) developed symptoms; case 3 (adult member of family) was admitted at the time of symptom onset per above	High (direct contact with skin/mucous membranes; no FFP3 respirator), intermediate (not specified), low (physical contact with appropriate PPE)	Symptom monitoring; low risk: passive surveillance; intermediate or high risk: active surveillance daily	No. of exposed HCP not provided; no transmissions outside the household were reported.	Hobson et al^18^

Note. HCP, healthcare personnel; HCF, healthcare facility; AIIR, airborne infection isolation room; PEP, postexposure prophylaxis with vaccine; ED, emergency department; CDC, Centers for Disease Control and Prevention; PPE, personal protective equipment; FFP3 respirator, filtering facepiece respirator class P3.

Definitions of exposures varied across reports, as did descriptions of personal protective equipment (PPE) used and risk stratification of exposed HCP. Symptom monitoring, both active and passive, was the most employed method to detect infection following exposure, and one study employed serological analysis in a subset of HCP. Postexposure prophylaxis (PEP) with vaccine was offered and administered in a subset of exposures. A single case of healthcare-associated transmission was described in an HCP in the United Kingdom. This exposure was deemed high risk due to changing presumably contaminated bedding while wearing disposable apron and gloves but not a face mask or respirator, during a period when the patient had active lesions prior to isolation. This HCP received PEP with live, attenuated vaccinia virus (Modified Vaccinia Ankara, MVA-BN, Bavarian Nordic, marketed as IMVANEX in Europe, JYNNEOS in the United States, and IMVAMUNE in Canada) 5–7 days after multiple exposures and developed illness 8 days after receiving vaccine.^4^

## Discussion

We undertook a rapid review of the literature to characterize, in nonendemic countries, the reported risk of patient-to-HCP exposure and transmission in healthcare facilities. Despite documented exposures in such settings, only a single transmission event has been reported. These findings are subject to important limitations, including variable definitions of exposure, rendering it difficult to quantify exposed HCP across published reports or to ascertain risk stratification among those exposed. Additionally, the granular details of each exposure (PPE worn by source and exposed, types of interactions that took place, and duration) are not available. Contact tracing and exposure investigations are resource intensive and rely upon potentially imperfect recollection from interviewed HCP. These practical challenges hinder the collection of data necessary to stratify risk and to comprehend more fully the nature of exposures in healthcare settings.

Proposed exposure definitions, which encompass both healthcare and nonhealthcare settings, have been developed by the UK Health Security Agency^5^ and the US Centers for Disease Control and Prevention.^6^ These definitions are not concordant in terms of risk stratification, recommendations for PEP, or work restrictions for HCP. Consensus definitions would allow not only for improved understanding of risk of exposure from specific interactions and modifiers of risk but also for comparisons across countries investigating exposures. Any exposure framework must include specific definitions of degree of risk based on the nature of source-exposed interactions (ie, direct or indirect contact, intact vs nonintact skin, mucous membranes) and the PPE worn by both the source (eg, face mask) and those exposed (eg, gown, gloves, face mask, N95 respirator or equivalent, eye protection).

In summary, based on published reports prior to the May 2022 global outbreak of monkeypox in nonendemic countries, the risk of exposure in well-resourced healthcare settings leading to transmission is low, with a single reported transmission event in the current literature. These findings may inform the provision of pre-exposure prophylaxis (PrEP), which has been recommended not only for laboratory personnel working with or performing diagnostic testing for orthopoxviruses and HCP who administer ACAM2000 (Smallpox [Vaccinia] Vaccine, Live) but also for HCP designated by public health authorities as response team members and for HCP who care for patients infected with orthopoxviruses.^7^ The literature, however, is limited both in scale and in the details required to effectively categorize risk. Evaluations of nosocomial exposures during the current outbreak may provide additional information regarding the risk of exposure in healthcare facilities, which would in turn provide information on both PrEP and PEP strategies, though comparisons across care settings will likely be hindered by inconsistent exposure definitions and risk classification.
